# Stapled Peptides as HIF‐1α/p300 Inhibitors: Helicity Enhancement in the Bound State Increases Inhibitory Potency

**DOI:** 10.1002/chem.202000417

**Published:** 2020-05-26

**Authors:** Kristina Hetherington, Zsofia Hegedus, Thomas A. Edwards, Richard B. Sessions, Adam Nelson, Andrew J. Wilson

**Affiliations:** ^1^ School of Chemistry University of Leeds Woodhouse Lane Leeds LS2 9JT UK; ^2^ Astbury Centre for Structural Molecular Biology University of Leeds Woodhouse Lane Leeds LS2 9JT UK; ^3^ School of Molecular and Cellular Biology University of Leeds Woodhouse Lane Leeds LS2 9JT UK; ^4^ School of Biochemistry University of Bristol Medical Sciences Building, University Walk Bristol BS8 1TD UK; ^5^ BrisSynBio University of Bristol, Life Sciences Building Tyndall Avenue Bristol BS8 1TQ UK

**Keywords:** chemical biology, oncology, peptidomimetics, protein–protein interactions, stapled peptides

## Abstract

Protein–protein interactions (PPIs) control virtually all cellular processes and have thus emerged as potential targets for development of molecular therapeutics. Peptide‐based inhibitors of PPIs are attractive given that they offer recognition potency and selectivity features that are ideal for function, yet, they do not predominantly populate the bioactive conformation, frequently suffer from poor cellular uptake and are easily degraded, for example, by proteases. The constraint of peptides in a bioactive conformation has emerged as a promising strategy to mitigate against these liabilities. In this work, using peptides derived from hypoxia‐inducible factor 1 (HIF‐1α) together with dibromomaleimide stapling, we identify constrained peptide inhibitors of the HIF‐1α/p300 interaction that are more potent than their unconstrained sequences. Contrary to expectation, the increased potency does not correlate with an increased population of an α‐helical conformation in the unbound state as demonstrated by experimental circular dichroism analysis. Rather, the ability of the peptide to adopt a bioactive α‐helical conformation in the p300 bound state is better supported in the constrained variant as demonstrated by molecular dynamics simulations and circular dichroism difference spectra.

## Graphical abstract

**Hold tight**: *S*,*S*‐Maleimide constrained HIF‐1α peptides are shown to be more potent inhibitors of the HIF‐1α/p300 interaction than their unconstrained counterparts due to stabilization of the bioactive conformation when bound to p300, as demonstrated by molecular dynamics simulations and difference circular dichroism (CD) spectroscopy.

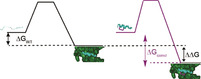

## Introduction

Protein–protein interactions (PPIs) control virtually all cellular processes and therefore regulate healthy and disease biology.^1^, ^2^, ^3^ For instance, in cancer, overexpression of pro‐survival BCL‐2 family proteins attenuates apoptosis supporting survival,^4^, ^5^ whilst the pattern recognition of toll‐like receptors plays a central role in inflammatory response.^6^, ^7^ Perturbation of PPIs using synthetic reagents is therefore desirable.^1^, ^2^, ^3^ In some cases lead discovery campaigns have successfully led to development of molecular therapeutics targeting PPIs.^4^ In particular, α‐helix mediated PPIs^8^ have received attention, in part due to the defined structural component of binding conferred by docking of an α‐helix from one protein into a cleft on another, but also as a consequence of the regular pattern of hot‐spot residues that are characteristic of such PPIs.^9^, ^10^, ^11^ This has facilitated the development of constrained peptide^12^, ^13^, ^14^, ^15^ and foldamer based^16^, ^17^, ^18^ topological mimics of the helix (i.e., mimicking the local conformation of the helix), alongside topographical mimics^19^, ^20^, ^21^, ^22^, ^23^ of the helix (i.e., mimicking the surface features of the helix).

Peptide “stapling” has been developed as a versatile method for targeting α‐helix mediated PPIs as well as those mediated by irregular binding interfaces.^12^, ^13^, ^14^, ^15^ Constraining a peptide in an α‐helical conformation has been shown to increase peptide proteolytic stability,^24^, ^25^ improve cellular uptake,^26^, ^27^, ^28^ and enhance target binding affinity through pre‐organization of the bioactive conformation,^29^, ^30^ The toolbox for constraining peptides into a bioactive (helical) conformation includes: hydrocarbon “staples”,^31^, ^32^ lactam bridges,^33^, ^34^, ^35^ Cu^I^‐catalysed azide‐alkyne cycloaddition,^36^, ^37^, ^38^, ^39^, ^40^ hydrogen‐bond surrogates,^41^, ^42^ and other chemistries.^43^, ^44^ Adding to this toolbox, the use of, simple disulfide bridges, crosslinking of (homo)cysteine residues and other modifications of thiols have been described.^15^, ^45^, ^46^, ^47^, ^48^, ^49^, ^50^, ^51^ We recently introduced, a stapling protocol that exploits the reaction of dibromomaleimide^52^, ^53^ with two (homo)cysteine residues, appropriately placed at *i,* and *i+4*, positions in the sequence.^49^, ^54^ The approach is versatile, proceeds in buffer on unprotected peptides, reversible, and does not require unnatural amino acids so may in principle be applied to commercially sourced peptides.

In this work, we apply dibromomaleimide stapling to the development of constrained peptide inhibitors of the HIF‐1α/p300 interaction—a promising but challenging target for anticancer drug‐development.^55^, ^56^ Using a combination of fluorescence anisotropy competition assays, experimental circular dichroism and molecular dynamics simulations, we show that when suitably positioned, introduction of the *S*,*S*‐maleimide crosslink within a 14 residue sequence from the C‐terminal region of HIF‐1α (residues 812–826) does not lead to a significant increase in helicity of the unbound peptide, yet this modification does increase inhibitory potency. The effect can be rationalised using molecular dynamics simulations and difference circular dichroism (CD) spectroscopy, which show that introduction of the constraint in the HIF‐1α peptide leads to better maintenance of the bioactive conformation when bound to p300. The results highlight the importance of considering the conformational stability of the bound state in designing constrained peptides as inhibitors of PPIs.

## Results and Discussion

The HIF‐1α/p300 PPI plays a key role in tumour metabolism and thus represents a promising anticancer target.^55^, ^56^ HIF‐1α is a transcription factor that regulates cellular response to hypoxia. When oxygen levels in the tissue are normal, HIF‐1α is degraded rapidly.^57^ However, under hypoxic conditions HIF‐1α translocates to the nucleus where it forms a heterodimer with HIF‐1β and this complex recruits the p300 transcriptional coactivator.^58^ The hypoxic response cascade results in expression of multiple genes to relieve oxygen deprivation. As such, cancer exploits this pathway to resupply oxygen to growing tumours.^59^, ^60^ The large interaction surface area comprising three helical regions over which binding free energy is dispersed render the HIF‐1α/p300 interaction challenging for inhibitor identification and design.^55^ Epidithioketopiperazine containing molecules such as Chetomin^61^ have been reported as HIF‐1α/p300 inhibitor molecules,^62^ however zinc ejection from p300,^63^ has been shown to contribute to the molecular mode of action, which may lead to nonspecific effects. Several small‐molecules have been identified as potential HIF‐1α inhibitors whilst a range of constrained peptide and helix mimetic inhibitors have also been described.^42^, ^64^, ^65^, ^66^

### Design and synthesis of constrained HIF‐1α peptides

**Figure 1:**
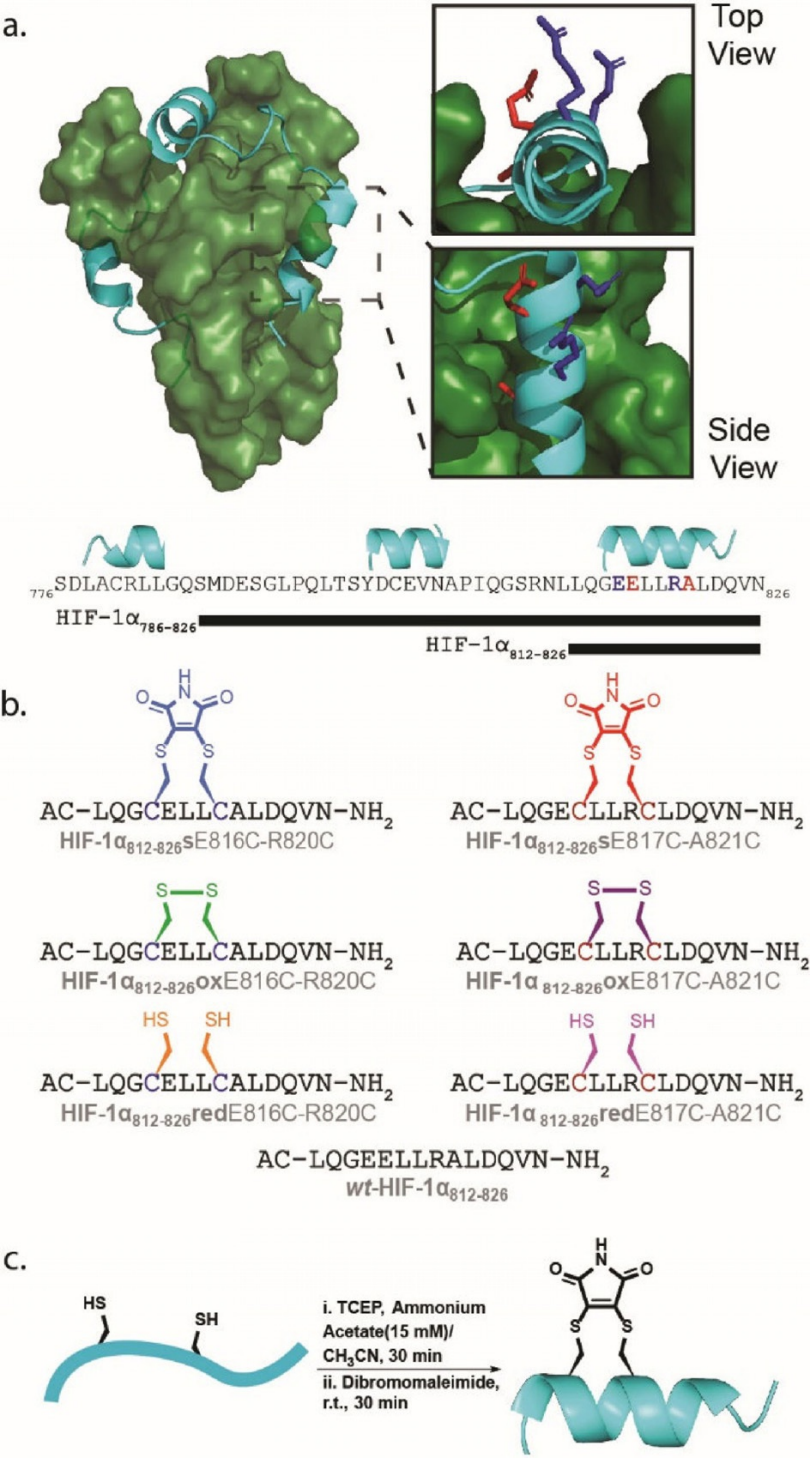
Design and synthesis of constrained HIF‐1α peptides as HIF‐1α/p300 inhibitors; a) HIF‐1α/p300 NMR solution structure (PDB:1L8C, p300 in forest green, HIF‐1α in cyan) with expansion (right) illustrating helix 3 (residues 812*–*826) and residues which were substituted for cysteine and subjected to stapling using dibromomaleimide, b) primary structure of the two HIF‐1α_**812***–***826**_ **s**E816C‐R820C and HIF‐1α_**812***–***826**_ **s**E817C‐A821C dibromomaleimide stapled variants: primary structures of dibromomaleimide (**s**), Oxidised (**ox**) and reduced (**red**) together with wild‐type (wt) sequence wt‐ HIF‐1α_**812***–***826**_, c) generic reaction scheme for preparation of dibromomaleimide stapled peptides and idealised helical conformation adopted as a consequence of stapling.

HIF‐1α binds to p300 by wrapping around it through three connected helical regions.^[67, 68]^ Amino acids within the second helix (residues 794–804) and third helix (residues 812–826) have been identified as important for binding to p300 while truncation of the third helix, the longest of the helical sequences, resulted in attenuation of p300 binding.^41^, ^65^, ^69^, ^70^, ^71^, ^72^, ^73^ Previously, our group explored the HIF‐1α/p300 PPI interface using truncated peptides, phage display of linear peptides, and, non‐antibody binding proteins (Affimer).^74^ Collectively these analyses ascertained that the second and third helical regions (HIF‐1α_794‐826_) of the 42 residue p300 C‐terminal transactivation domain (TAD) were sufficient for p300 binding. These data informed our choice in this work, to introduce a constraint in the third, C‐terminal, helix: HIF‐1α_821–826_ (Figure 1).

Cysteine residues were placed at *i* and *i+4* positions replacing E816 and R820, or E817 and A821 to explore two distinct stapling locations (Figure 1 a). Alongside the wild‐type peptide (**wt**), the dibromo maleimide stapled (**s**), oxidised disulfide (**ox**) and reduced thiol (**red**) peptides for each variant were prepared (Figure 1 b). The peptides were prepared using Fmoc solid phase peptide synthesis on Rink Amide MBHA resin and capped with acetyl groups at the N‐terminus. After purification, the peptides were subjected to 30 minutes of stirring with TCEP to ensure reduction of any disulfide, followed by the addition of dibromomaleimide for 30 minutes (Figure 1 c), representing a reduction on the stapling reaction time we described in prior work.^49^ Fully oxidised (**ox**) and fully reduced (**red**) variants of the peptides were also obtained for biophysical analyses together with the native sequence.

### Biophysical analyses of constrained HIF‐1α peptides

The effect of stapling on inhibition of the HIF‐1α/p300 interaction was then assessed using fluorescence anisotropy‐based (FA) competition assays. A full‐length HIF‐1α peptide was used as the tracer ligand: FITC‐Ahx‐HIF‐1α_**786–826**_, *K*_d_=10.5(±4.4) nm (Figure S1 ESI). None of the HIF‐1α_812–826_ E817C‐A821C sequences (e.g., Figure 1 b red staple), was observed to displace the tracer in the competition assays up to a concentration of 0.9 mm (Figure S2 ESI). This was not unexpected given that E817 and A821 point towards the p300 protein interface in the NMR structure of the HIF‐1α/p300 complex (Figure 1 a residues in red); thus a staple at this location likely introduces a steric clash with the p300 surface. The E817C‐A821C series of peptides was thus not studied further although CD analyses indicated minimal differences in helicity across this series of peptides (Figure S3 ESI).

**Figure 2:**
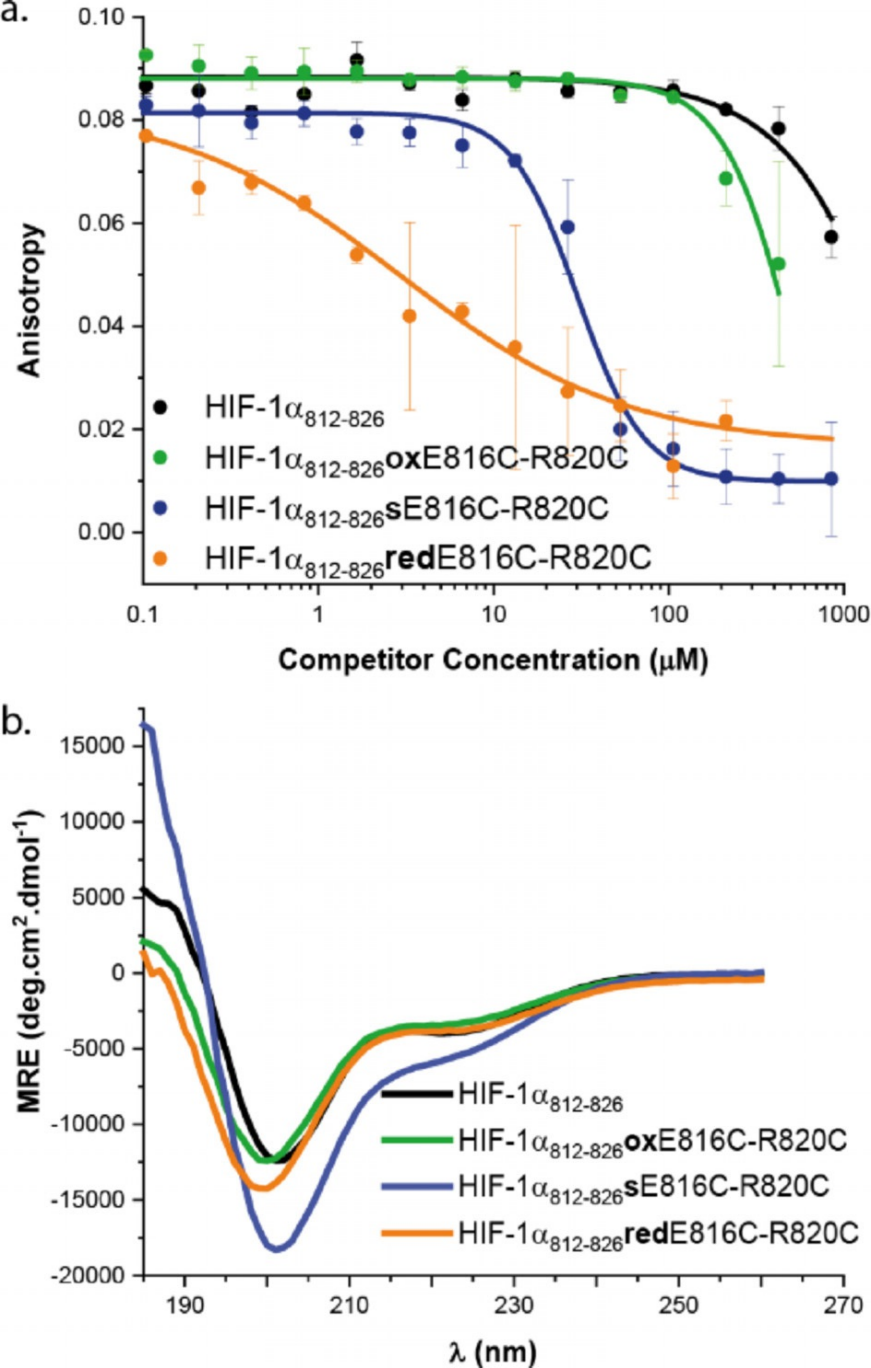
Biophysical characterization a) FA data for the E816‐R820 peptide series, FITC‐Ahx‐HIF‐1α_786–826_ tracer (25 nm) p300 (100 nm) 20 mm Tris, 100 mm NaCl 0.1 mm DTT, pH 7.46. b) CD spectra for wt‐HIF‐1α_812–826_, HIF‐1α_**812***–***826**_ **s**E816C‐R820C, HIF1α_**812***–***826**_ **ox**E816C‐R820C and HIF‐1α_**812***–***826**_ **red**E816C‐R820C (250 μm concentration, 20 mm sodium phosphate, pH 7.55).

In contrast, the E816‐R820 series of peptides comprising the stapled HIF‐1α_**812**–**826**_ **s**E816C‐R820C, disulfide bridged HIF‐1α_**812**–**826**_ **ox**E816C‐R820C and the reduced thiol HIF‐1α_**812**–**826**_ **red**E816C‐R820C peptide gave more interesting results. The ability of this series of peptides to outcompete the FITC‐Ahx‐HIF‐1α_**786**–**826**_ tracer in an FA competition assay was assessed and also compared to the wild‐type sequence wt‐HIF‐1α_**812**–**826**_ (Figure 2 a). To confirm the peptides were still in their respective reduced or oxidised states, HRMS data were collected following the assay (Figure S4 ESI). The wt‐HIF‐1α_812–826_ gave weak inhibition and could not fully displace tracer consistent with prior results.^74^ Similarly, HIF‐1α_**812**–**826**_ **ox**E816C‐R820C also showed weak inhibitory potency with an IC_50_ >600 μm. However, HIF‐1α_**812**–**826**_ **s**E816C‐R820C demonstrated considerably improved inhibitory potency with IC_50_ = 30.3(±5) μm (Figure 2). Unexpectedly, HIF‐1α_**812**–**826**_ **red**E816C‐R820C, was also shown to act as an inhibitor of the interaction with an IC_50_ = 9.9(±0.6) μm (see below).

A significant number of studies have shown that introduction of judiciously placed constraints biases the conformation of peptides in a helical conformation and this can result in enhanced target binding affinity and/or inhibitory potency arising presumably due to the reduced entropic cost of binding.^12^, ^13^, ^29^ Indeed our previous studies on BCL‐2 family variant peptides constrained through maleimide bridging of *i* and *i+*4 bis(homo)cysteines demonstrated both enhanced helicity and inhibitory potency towards PPIs of MCL‐1 and BCL‐x_L_.^49^ To assess the extent to which preorganization of the peptide ligand correlated with inhibitory potency, we carried out circular dichroism analyses on the HIF‐1α variant peptides. Surprisingly, the CD spectra (Figure 2 b) showed limited differences between variants, with all adopting a predominantly random coil conformation and only HIF‐1α_**812**–**826**_ **s**E816C‐R820C ([*θ*]_MRE‐222_ −6658, 20 % helicity) showing a higher helicity than the other variants (wt‐HIF‐1α_812826_; [*θ*]_MRE‐222_ −3954, 12 % helicity, HIF‐1α_**812**–**826**_ **ox**E816C‐R820C; [*θ*]_MRE‐222_ −3766, 11 % helicity, HIF‐1α_**812 826**_ **red**E816C‐R820C; [*θ*]_MRE‐222_ −3375, 10 % helicity). The difference in helicity between HIF‐1α_**812**–**826**_ **s**E816C‐R820C and the other variants is energetically small; where the binding mechanism occurs via conformational selection this difference could make a small contribution to differences in inhibitory potency (see later). We also performed thermal unfolding experiments on the isolated peptides (Figure S5 ESI). The spectrum of wt‐HIF‐1α_812–826_ sequence showed little variation in temperature. A more significant response was observed for the constrained variants, HIF‐1α_**812**–**826**_ **s**E816C‐R820C, disulfide bridged HIF‐1α_**812**–**826**_ **ox**E816C‐R820C indicating greater variation in structure with increasing temperature.

The shallow inhibitory curve for HIF‐1α_**812**–**826**_ **red**E816C‐R820C indicated a non‐simple and potentially nonspecific mode of inhibition. We hypothesised that the free thiols may be detrimental to binding. As such, circular dichroism thermal denaturation experiments were performed on p300 in isolation and in the presence of stoichiometric HIF‐1α_**812**–**826**_ **s**E816C‐R820C or HIF‐1α_**812**–**826**_ **red**E816C‐R820C ligands (Figure S6 ESI). The thermal CD behaviour of p300 with HIF‐1α_**812**–**826**_ **red**E816C‐R820C was dramatically different; the sample had a markedly reduced MRE at 222 nm and minimal differences were observed on increasing temperature (Figure S7 ESI). Such a significant reduction in p300 helicity upon addition of HIF‐1α_**812**–**826**_ **red**E816C‐R820C is consistent with a loss of p300 structural integrity and nonspecific binding, providing an explanation for the observed dose response behaviour. Chetomin, a previously discovered HIF‐1α/p300 inhibitor,^61^ which contains an electrophilic disulfide was shown to act as a nonspecific inhibitor by zinc ejection^63^ whilst several other reagents have subsequently been shown to elicit similar nonspecific p300 binding through cysteine modification and/or zinc ejection.^75^ The observation here that bisthiol containing ligands can also have nonspecific binding will be the focus of future studies, nonetheless, this adds to the palate of functionality that can confer inherently unselective p300 binding.

### Molecular dynamics (MD) analyses and difference circular dichroisms (CD) of constrained HIF‐1α peptides

**Figure 3:**
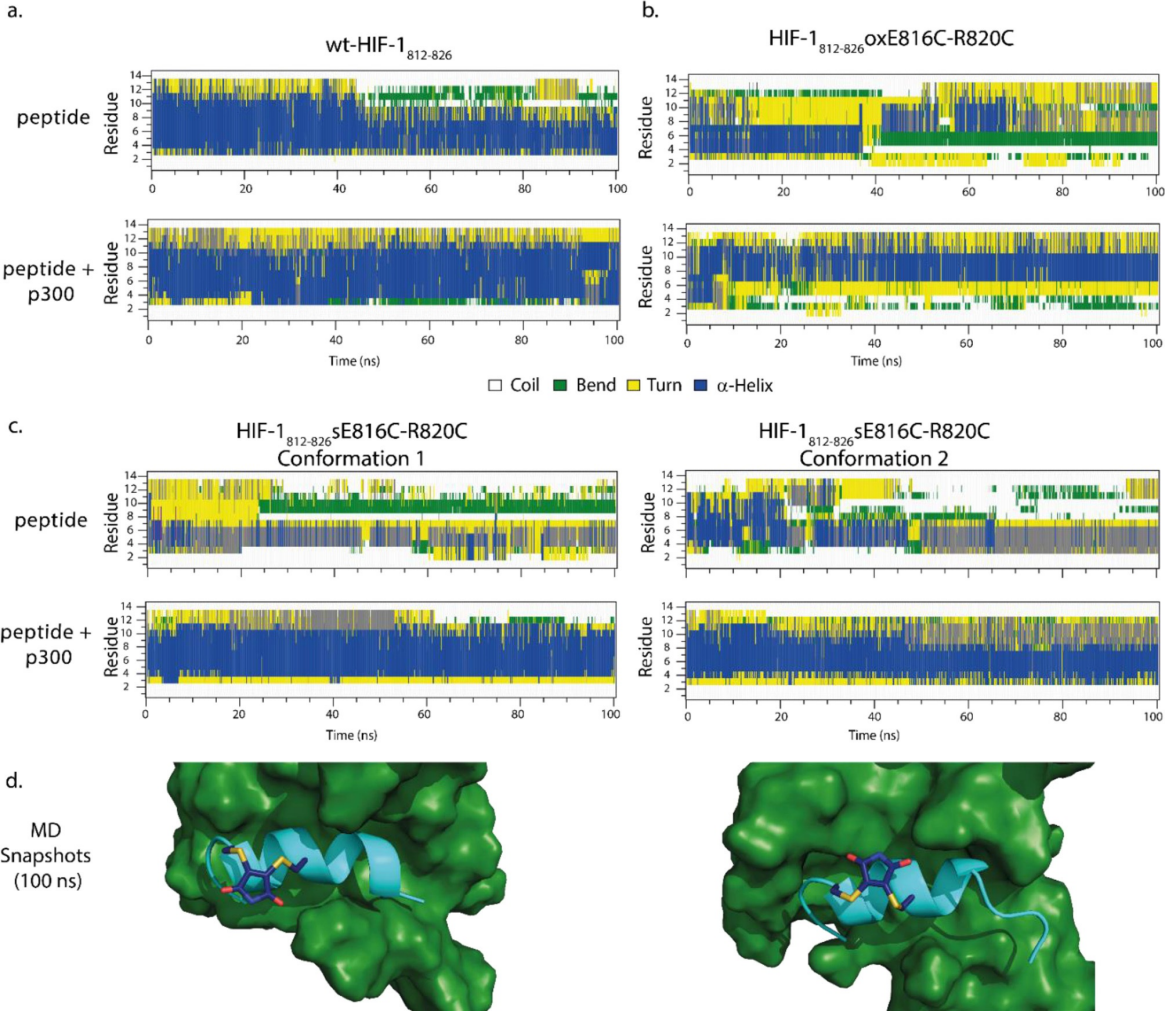
Conformational analyses of HIF‐1α_**812***–***826**_ **s**E816C‐R820C: a) 100 ns MD simulation of wt*‐*HIF‐1α_**812***–***826**_ peptide in the absence (top panel) and presence (bottom panel) of p300, b) MD simulations of the HIF‐1α_**812***–***826**_ **ox**E816C‐R820C peptide (panels to the right) in the absence (top panel) and presence (bottom panel) of p300, c) MD simulations of HIF‐1α_**812***–***826**_ **s**E816C‐R820C in isolation and in complex with p300 in the two dominant conformations adopted, d) MD snapshot of two stable p300 bound conformations of HIF‐1α_**812***–***826**_ **s**E816C‐R820C.

Given the conformation of the peptides in the absence of p300 varied minimally, we sought an alternative explanation for the significantly enhanced potency of FITC‐Ahx‐ HIF‐1α_**786**–**826**_/p300 inhibition by HIF‐1α_812–826_ **s**E816C‐R820C in comparison to the other HIF‐1α variant peptides. First, we performed MD simulations of the peptides in solution and in complex with p300 over a 100 ns timeframe (percentage helicities averaged using the final 50 ns). The MD simulations indicated that wt‐HIF‐1α_812–826_ has a significant helical conformation (43 %) and moderate increase in helicity (61 %) when in complex with p300 (Figure 3 a). The MD simulation for wt‐HIF‐1α_812–826_ in isolation contrasts somewhat with conclusions obtained from solution CD spectra (Figure 2 b) which indicate limited population of the helical conformation in isolation. It should be noted that we use the MD simulations here to provide a more qualitative evaluation of changes in the preferred conformation between bound and unbound states, rather than absolute energetic differences so caution should be exercised in making a direct comparison with the CD data, in particular between each of the three peptide ligands. The HIF‐1α_**812**–**826**_ **ox**E816C‐R820C peptide displayed low helicity (21 %) and a moderate increase (37 %) when in complex with p300 about the stapled position (Figure 3 b). Finally, two dominant conformers were observed for HIF‐1α_**812**–**826**_ **s**E816C‐R820C over the course of the simulation with the maleimide bridge oriented in opposing directions with respect to the helix (Figure 3 d). Both had little helical character (24 % conformer 1 and 29 % conformer 2), with the most helical region observed across the first six residues of the sequence, in agreement with the CD data (Figure 3 c). In complex with p300 a more dramatic increase in helicity was observed (56 % and 54 %, respectively); both the HIF‐1α_**812**–**826**_ **s**E816C‐R820C conformers held a helical conformation propagated over a greater number of residues in the sequence for a greater portion of the simulation when compared to the unbound situation. Although constrained peptides have been shown to have enhanced protein binding affinity as a consequence of interaction between protein and staple,^76^, ^77^ that does not appear to be the case here and instead the results suggest that the higher affinity of HIF‐1α_812–826_ **s**E816C‐R820C arises in part as a consequence of stabilizing the p300 bound conformation of the peptide ligand.

**Figure 4:**
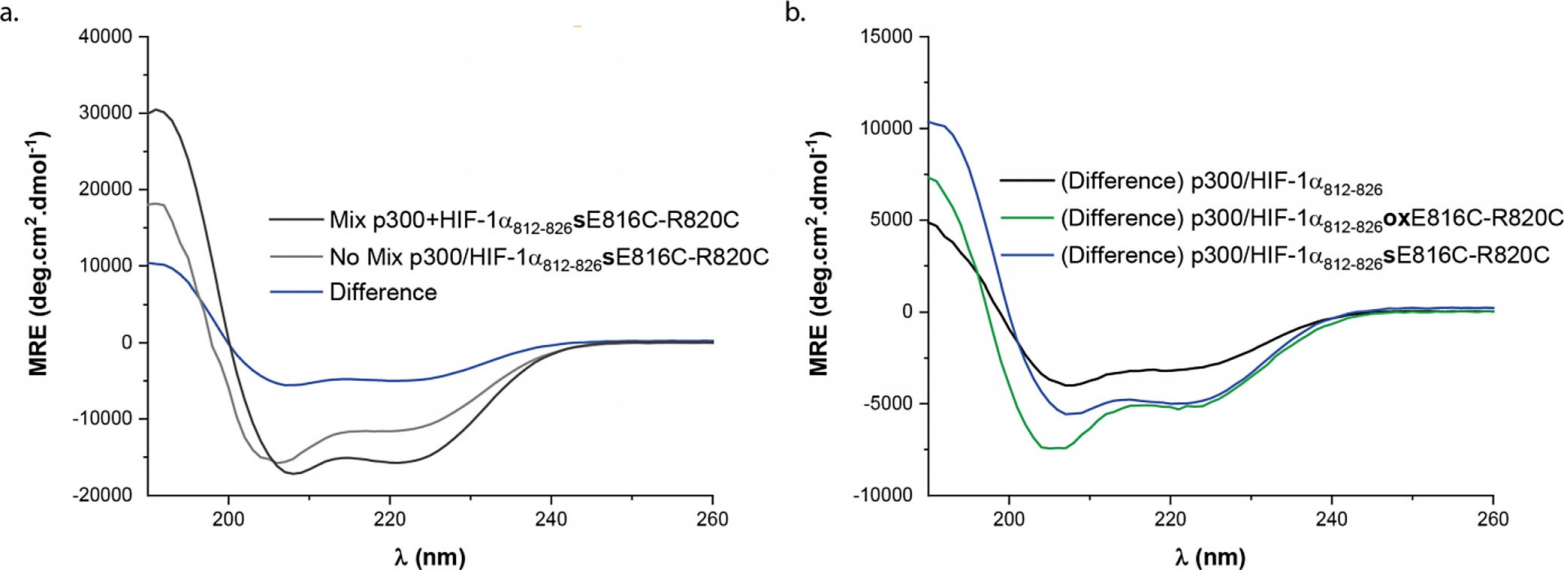
CD analysis: a) CD data for the additive (grey), combined (black) and difference spectra (blue) for the HIF‐1α_**812***–***826**_ **s**E816C‐R820C variant peptide in the presence of p300. b) Difference CD: spectra for binding of wt‐HIF‐1α_812–826_, (black) stapled peptide, HIF‐1α_**812***–***826**_ **s**E816C‐R820C (blue) and HIF‐1α_**812***–***826**_ **ox**E816C‐R820C (green) to p300.

To support the MD simulations, we obtained CD difference spectra. The difference spectra was obtained by subtracting the additive spectra of the peptide and protein acquired separately from a sample containing a mixture of the two combined (Figure 4 a CD data acquired for p300 and HIF‐1α_**812**–**826**_ **s**E816C‐R820C)_._ It was reasoned that the difference between the additive CD signal and the CD signal of the combined sample—where the protein and peptide should be interacting—would offer insight on the influence of p300 on the conformation of the HIF‐1α peptides and reaffirm a binding event. This difference was plotted as mean residue ellipticity versus wavelength (Figure 4 b). The experiment was performed at a concentration of 40 μm, close to the IC_50_ value for the stapled HIF‐1α_812–826_ **s**E816C‐R820C peptide. Firstly, the outcome of the experiment confirms direct interaction between stapled peptide and protein target. Secondly, for HIF‐1α_**812**–**826**_ **s**E816C‐R820C, the difference spectrum is consistent with an idealized α‐helical conformation with minima at 208 and 222 nm.^78^, ^79^ The MD simulations do not suggest the constraint makes significant non‐covalent contacts with p300, whilst conformational changes in p300 that increase helicity in p300 (relative to the largely helical apo p300) are unlikely to lead to such a significant difference in mean residue ellipticity. Therefore, the substantial increase in MRE in the difference spectra is consistent with a significant increase in helical stability of the peptide ligand in the bound state supporting the conclusions of the MD simulation. The difference spectrum for the wt‐HIF‐1α_812–826_ is weaker, consistent with the MD simulations that imply minimal change in helicity upon binding. Lastly, the difference spectrum for HIF‐1α_812–826_ **ox**E816C‐R820C, also indicated an increase in helicity, however the minima at ≈204 nm present for the unbound peptide persisted, suggesting that this peptide is unable to adopt an idealized and fully helical conformation in the bound state. Again, these data are consistent with the conclusions of the MD analyses.

## Conclusions

In summary we have designed, synthesised and tested *S*,*S* maleimide constrained HIF‐1α peptides as HIF‐1α/p300 inhibitors. The constrained peptide HIF‐1α_**812**–**826**_ **s**E816C‐R820C, showed improved binding affinity but moderate increase in helicity in the unbound from. In contrast MD simulations were able to show that the p300 bound form of the peptide adopts a more stable helix as a consequence of introducing the staple. The inhibitory potency of the best ligand developed in this study (for HIF‐1α_**812**–**826**_ **s**E816C‐R820C at IC_50_ ≈30 μm) although greater than one order of magnitude superior to the wild‐type or disulfide variants indicates further optimization will be required to develop chemical probes. Previously, MD simulations of eIF4G peptides demonstrated that conformational constraint with hydrocarbon linkages perturbed the structural dynamics of peptides when bound or unbound to eIF4E.^80^ These MD simulations revealed that whilst stabilization of an unbound peptide in a helical conformation can readily be achieved, this could adversely affect binding affinity by favouring metastable conformations that incur a reorganizational penalty on target engagement, or preventing key side‐chains from adopting the orientation required for binding. They also demonstrated that the combination of a constraint and judicious sequence modification promoted solution conformations that matched the ideal bound conformation. A related observation was recently described in studies on constrained inhibitors of transcription factor assembly where subtle changes to structure were observed to affect the stability of the bound state.^81^ The results presented here for HIF‐1α_**812**–**826**_ **s**E816C‐R820C differ; whilst a moderate increase in helicity might make a contribution to enhanced p300 binding and ligand induced changes in p300 conformation cannot be discounted, our data reveal the potential to enhance target binding affinity of constrained peptides by explicit stabilization of a bound conformation (Figure S8). The results add to the complex effects on molecular recognition that can arise upon constraining a peptide which include enthalpy‐entropy compensation, induced‐fit recognition,^82^ modulating binding mechanism and dynamics.^83^, ^84^, ^85^, ^86^ Thus, our future studies will focus on collective application of these concepts to a more refined approach for the introduction of constraints in peptide ligands, and in particular future further optimization of peptidomimetic HIF‐1α/p300 inhibitors.

## Experimental Section

### Solid phase peptide synthesis

**General remarks**: All amino acids and resins were purchased from either Novabiochem (Merck) or Sigma–Aldrich. All amino acids were *N*‐Fmoc protected and side chains protected with Boc (Lys); O*t*Bu (Asp, Ser, Thr); Trt (Asn, Gln); Pbf (Arg). Synthesis of all peptides was performed using a microwave assisted automated peptide synthesiser (CEM, Liberty or Liberty Blue). DMF used in peptide synthesis was of ACS grade and from Sigma–Aldrich. Peptides were synthesised on an 0.1 mmol scale. Lyophilisation was performed using a BenchTop Pro with Omnitronics^TM^ from VirTis SP Scientific. Preparative HPLC was performed on an Agilent Technologies 1260 infinity controller in conjunction with a diode array detector. Analytical HPLC was performed on an Agilent Technologies 1260 infinity controller in conjunction with a diode array detector. Mass spectrometry data were obtained on a Bruker MaXis Impact using electrospray ionisation (ES)MS instruments as appropriate.

**Cycles for automated peptide synthesis**: Resin loading: Clean reaction vessel; wash with DMF, wash with CH_2_Cl_2_; transfer resin to reaction vessel; wash with DMF, wash with CH_2_Cl_2_; vessel draining.

**Deprotection and coupling**: Clean resin dip tube, wash with DMF (15 mL) add 20 % piperidine in DMF (6 mL), microwave method (30 s), wash with DMF (15 mL), clean resin dip tube, wash with DMF (15 mL), add amino acid (2.5 mL), add coupling reagent (1 mL), add activator base (0.5 mL), microwave method (5 min), wash with DMF (15 mL), drain.

For methods that ***did not*** use microwave assistance, the reaction cycle was the same, expect the microwave method for deprotection and coupling was replaced by agitation of the resin at rt for 10 min and 90 min, respectively.

After the final residue, the resin was ejected from the reaction vessel and linker coupling, capping, cleavage and deprotection was performed manually using methods A to B.

For the specific microwave methods used see Supporting Information.

**Method A**: N‐terminal acetylation: Acetic anhydride (10 equiv) and DIPEA (10 equiv) were dissolved in DMF (1 mL) and the solution was transferred to the resin. After 2 h, the resin was drained, washed with DMF (3×2 mL×2 min) and successful capping determined by a negative colour test (Method C).

**Method B**: Cleavage and deprotection of Rink amide MBHA resin: After elongation and N‐terminal capping was complete, the resin was washed with CH_2_Cl_2_ (5×2 mL×2 min), Et_2_O (5×2 mL×2 min) and dried under vacuum for about 2 h. Peptides were simultaneously cleaved and side‐chain deprotected using Reagent K TFA:EDT:thioanisole:phenol:H_2_O 82:3:5:5:5 (3×2 mL×2 h). The solution was precipitated in ice‐cold Et_2_O (25 mL) and placed in a centrifuge (3000 rpm×10 min), the supernatant removed and the precipitate resuspended in ice‐cold Et_2_O and placed in a centrifuge again. This process was repeated 3 or 4 times and the precipitate was dried under a stream of nitrogen overnight, before being dissolved in H_2_O and lyophilised.

**Peptide purification**: Crude peptides were suspended in H_2_O as concentrated as possible, fractions were checked by LCMS, concentrated in vacuo and lyophilised. Peptides were purified by preparative UV‐ or MD‐HPLC using a Jupiter Proteo preparative column (reversed phase) on an increasing gradient of acetonitrile in water + 0.1 % formic acid (v/v) at a flow rate of 10 mL min^−1^. Crude peptides were suspended in H_2_O at an approximate concentration of 20 mg mL^−1^. Purification runs injected a maximum of 5 mL of crude peptide solution and were allowed to run for 30 min, with acetonitrile increasing at a stated gradient. In regards to UV‐HPLC, the eluent was scanned with a diode array at 220, 210 and 280 nm. In regards to MD‐HPLC the mass directed chromatography software Masshunter by ChemStation (Agilent) was used to allow the collection of the desired peptide by mass, with the eluent split into an Agilent 6120 Quadropole LCMS which triggers collection of eluent at a programmed *m*/*z*. Fractions containing purified peptide were combined, concentrated in vacuo and lyophilised.

### Fluorescence anisotropy

Fluorescence anisotropy assays were performed in 384‐well plates (Greiner Bio‐one). Each experiment was run in triplicate and the fluorescence anisotropy measured using a Perkin–Elmer EnVisionTM 2103 MultiLabel plate reader, with excitation at 480 nm (30 nm bandwidth), polarised dichroic mirror at 505 nm and emission at 535 nm (40 nm bandwidth, S and P polarised). All assays were performed in 20 mm Tris, 100 mm NaCl 0.1 mm DTT, pH 7.46 unless otherwise stated and data analysed following previously published methods.

The data from both the P (perpendicular intensity) and S (parallel intensity) channels, resulting from this measurement and corrected by subtracting the corresponding control wells, were used to calculate the intensity and anisotropy for each well following Equations (1), (2):

(1) I=(2PG)+S

(2) r=(S-PG)

(3) $$L{}_{b}=\left(r-r{}_{\min}\right)/\lambda \left(r{}_{\max}-r\right)+r-r{}_{\min}$$

(4) $$y=\{\left(k+x+\left[\mathrm{FL}\right]\right)-\surd \left\{k+x+\left[\mathrm{FL}\right]{}^{2}-4\times \left[\mathrm{FL}\right]\right\}\}/2$$

Fluorescence anisotropy data were processed as described previously.^83^

*r*=anisotropy, *I*=total intensity, *P*=perpendicular intensity, *S*=parallel intensity, *G*=instrument factor which was set to 1 for all experiments, *L*_b_=fraction ligand bound, *λ*=*I*_bound_/*I*_unbound_=1, [FL]=concentration of fluorescent ligand, *k*=*K*_d_, *y*=*L*_b_* Flu‐trimer and *x*=[added titrant], *G* is an instrument gain factor.

Where *I* is the total intensity, *G* is an instrument factor which was set to 1 for all experiments and *r* is the anisotropy. The average anisotropy (across three experimental replicates) and the standard deviation of these values were then calculated and fit to a sigmoidal logistic model [Eq. (3)] using OriginPro 9.0 which provided the IC_50_ and error values: *y* = *r*_max_ + (*r*_min_−*r*_max_)/(1+(*x*/*x_o_*) *p*).

### Direct binding assay for FITC‐Ahx‐HIF‐1α_786–826_ tracer

Fluorescence anisotropy direct titration assays were performed with protein concentration diluted over 16–24 points using 1/2 dilutions. 20 μL of buffer was first added to each well. 20 μL of a solution of protein was added to the first column. The solution was well mixed and 20 μL was taken out and added to the next column and so on. This operation consists on serial dilution of the protein across the plate. Finally, 20 μL of tracer was added to the wells. For control wells, the tracer peptide was replaced with an identical volume of assay buffer and plates were read after 45 minutes.

### Competition binding assays

FA competition assays were performed in 384 well plates with the concentration of peptide competitor typically starting from 850 μm, diluted over 16 points in 1/2 regime with fixed protein and tracer concentrations. FITC‐HIF‐1α_786–826_ was added to each well to give a final concentration of 50 nm. For control wells, the tracer peptide was replaced with an identical volume of assay buffer. The total volume in each well was 60 μL. Plates were read after 45 minutes of incubation at room temperature.

### Circular dichroism

Spectra were recorded on a chirascan circular dichroism spectropolarimeter (Applied Photophysics), at 20 °C, using 1 mm cells and a scan speed of 5 nm min^−1^. The spectra were averaged over three repeats with a buffer baseline subtracted. Peptide concentrations of approximately 0.1 mg mL^−1^ were used (although the exact concentration was used to allow determination of MME).

**p300/peptide spectra and thermal unfolding**: Spectra were recorded on a chirascan circular dichroism spectropolarimeter (Applied Photophysics), from 20–90 °C, using 1 mm cells and a scan speed of 5 nm min^−1^. The spectra were averaged over three repeats with a buffer baseline subtracted. Protein concentrations of approximately 0.2 mg mL^−1^ were used (although the exact concentration was used to allow determination of MME). Peptide concentrations were also of approximately 0.2 mg mL^−1^, in a 1:1 ratio with the protein. The helical content of protein/peptide complex was determined from the mean residue ellipticity at 222 nm, [*θ*] (deg cm^2^ dmol^−1^) and compared to that of the protein on its own.

**p300/peptide difference spectra**: Spectra were recorded on a chirascan circular dichroism spectropolarimeter (Applied Photophysics), at 20 °C, using 10 mm cells and a scan speed of 5 nm min^−1^. The protein and peptide were each dissolved in buffer and added to a sample cell, separated by a partition. This prevented the two solutions from mixing while the CD would acquire a data set which showed an additive signal of the protein and peptide without these two interacting. Following the CD acquisition of the two separated solutions, the cuvette was given a shake and the two solutions mixed. CD spectra was acquired for the mixture. The spectra were averaged over three repeats with a buffer baseline subtracted. Each peptide would have three CD traces, one for the signal in the cuvette where the peptide and protein solution were separated (no mix), one of the mixed solutions (mix) and the CD trace of the difference between these two which was calculated by subtracting the raw data. This CD signals was plotted as mean residue ellipticity [*θ*] (deg cm^2^ dmol^−1^) versus wavelength.

### Molecular dynamics

Model 1 was taken from the PDB structure file 1L8C and the B subunit edited to leave residues L136 to N149 and the resulting peptide capped with N‐terminal acetyl and C‐terminal amido groups. Cysteine variations and the maleimide stapled peptides were built with Chimera v1.13.^87^ The protein was parameterised with the amber99SB‐ildn forcefield^88^ and the staples with GAFF.^89^ All simulations were performed using GROMACS v5.1.5^90^ using the following general protocol. Hydrogen atoms were added consistent with pH 7. The protein was placed in an orthorhombic box 2 nm larger than the protein in each dimension and filled with TIP3P water containing 0.15 m sodium chloride ions to give a charge‐neutral system overall. After 10 000 steps of steepest descent minimisation, molecular dynamics was initiated with random velocities while restraining the protein backbone to its original position with a force constant of 1000 kJ nm^−1^ for 0.2 ns. Simulations were developed for a further 100 without the backbone position restraints under periodic boundary conditions. The Particle Mesh Ewald's method was used for long range electrostatic interactions while short range Coulombic and van de Waals energies were truncated at 1.4 nm. The temperature was maintained at 300 K using the v‐rescale method and the pressure at 1 bar with the Berendsen barostat and a 2 fs time step for the leapfrog integrator. Bond constraints were implemented with the LINCS method and SETTLE used for waters. Trajectories were processed and analysed with the GROMACS tools and visualised with VMD 1.9.3.^91^

## Conflict of interest

The authors declare no conflict of interest.

## Supporting information

As a service to our authors and readers, this journal provides supporting information supplied by the authors. Such materials are peer reviewed and may be re‐organized for online delivery, but are not copy‐edited or typeset. Technical support issues arising from supporting information (other than missing files) should be addressed to the authors.

Supplementary
