# Supplementary material for: Stapled Peptides as HIF‐1α/p300 Inhibitors: Helicity Enhancement in the Bound State Increases Inhibitory Potency
Source: Chemistry. 2020 May 26;26(34):7638–46. doi: 10.1002/chem.202000417 (PMC7318359; doi:10.1002/chem.202000417)
Supplement: Supplementary file 1 — Supplementary [file CHEM-26-7638-s001.pdf]

Chemistry–A European Journal

Supporting Information

Stapled Peptides as HIF-1α/p300 Inhibitors: Helicity Enhancement in the Bound State Increases Inhibitory Potency

Kristina Hetherington,[a, b] Zsofia Hegedus,[a, b] Thomas A. Edwards,[b, c] Richard B. Sessions,[d, e] Adam Nelson,[a, b] and Andrew J. Wilson*[a, b]

Supplementary Figures

HIF-1α full length sequence, tracer sequence (HIF-1α$_{786\text{-}826}$) and helix3 sequence (HIF-1α$_{812\text{-}826}$).

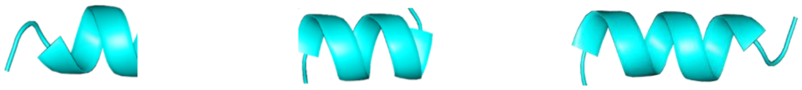

$_{776}$SDLACRLLGQSMDESGLPQLTSYDCEVNAPIQGSRNLLQGEELLRALDQVN$_{826}$

$_{786}$SMDESGLPQLTSYDCEVNAPIQGSRNLLQGEELLRALDQVN$_{826}$ **HIF-1α$_{786\text{-}826}$**

$_{812}$LQGEELLRALDQVN$_{826}$ **HIF-1α$_{812\text{-}826}$**

FITC-Ahx-HIF-1α$_{786\text{-}826}$ Tracer Assay data:

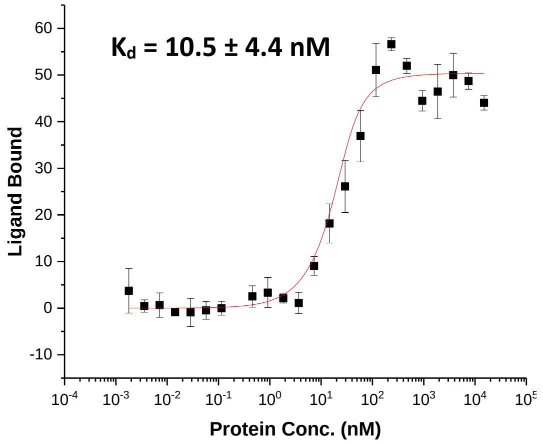

Figure S1. Direct titration assay **FITC-Ahx-HIF-1α$_{786\text{-}826}$** (25 nM) p300 (15 µM), 20 mM Tris, 100 mM NaCl 0.1mM DTT, pH 7.46

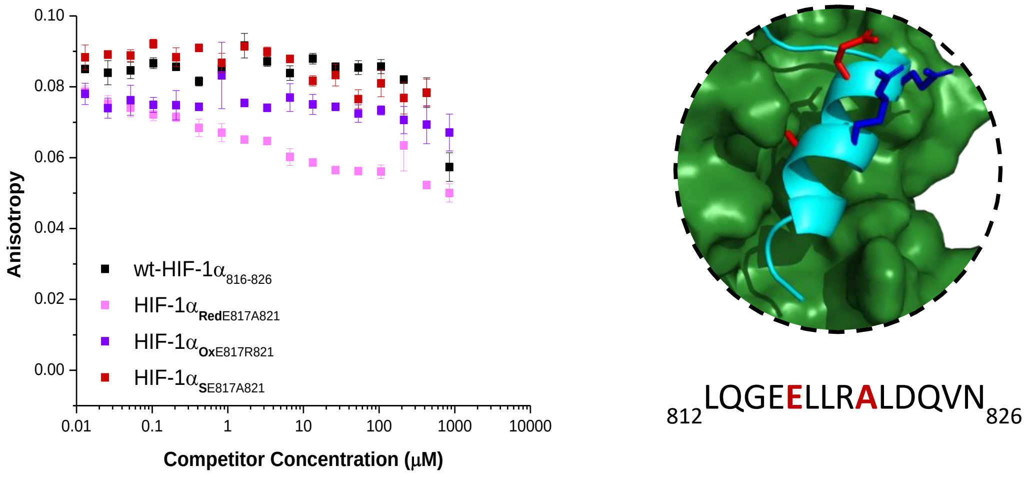

Figure S2. FA competition assay for HIF-1α$_{SE817A821}$, FITC-Ahx-HIF-1α$_{786\text{-}826}$ (25 nM), p300 (100 nM) 20 mM Tris, 100 mM NaCl 0.1mM DTT, pH 7.46

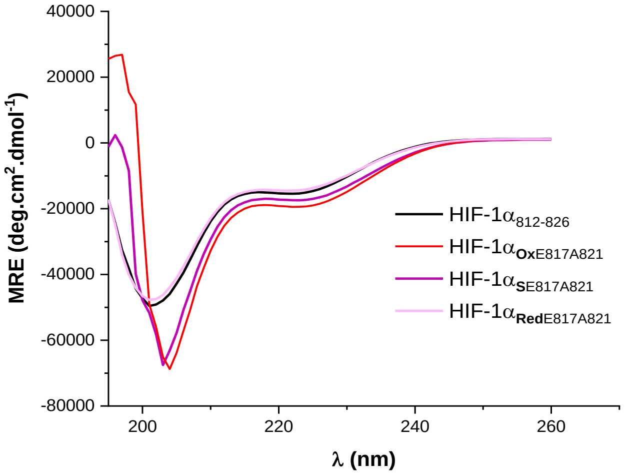

Figure S3. CD data for the E817C-A821C series of peptides

HIF-1α$_{812\text{-}826}$redE816C-R820C

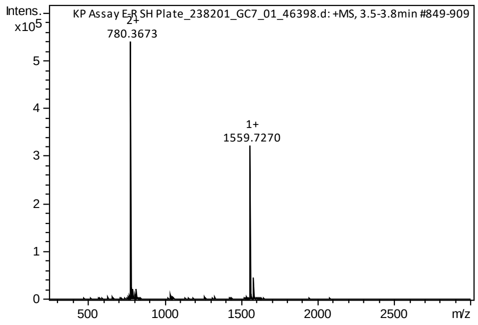

HIF-1α$_{812\text{-}826}$oxE816C-R820C

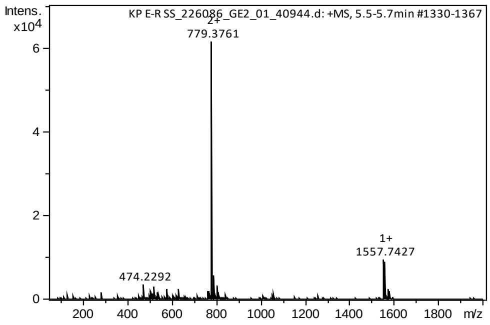

HIF-1α$_{812\text{-}826}$redE817C-A821C

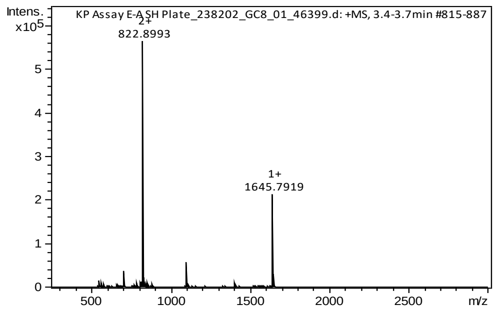

HIF-1α$_{812\text{-}826}$oxE817C-A821C

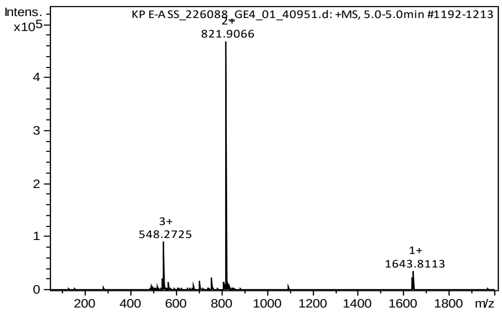

Figure S4. HRMS of assay wells to confirm reduced and oxidised state of peptides still present

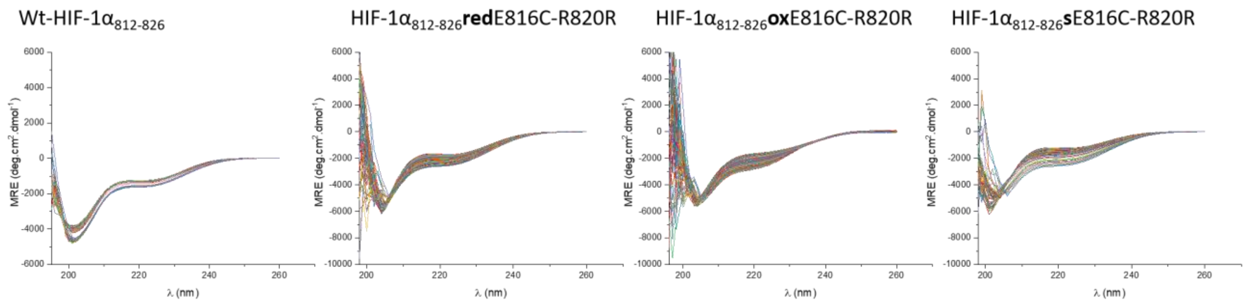

Figure S5. CD thermal melting experiment of peptides at 250 µM concentration, in 20 mM Phosphate, pH 7.55

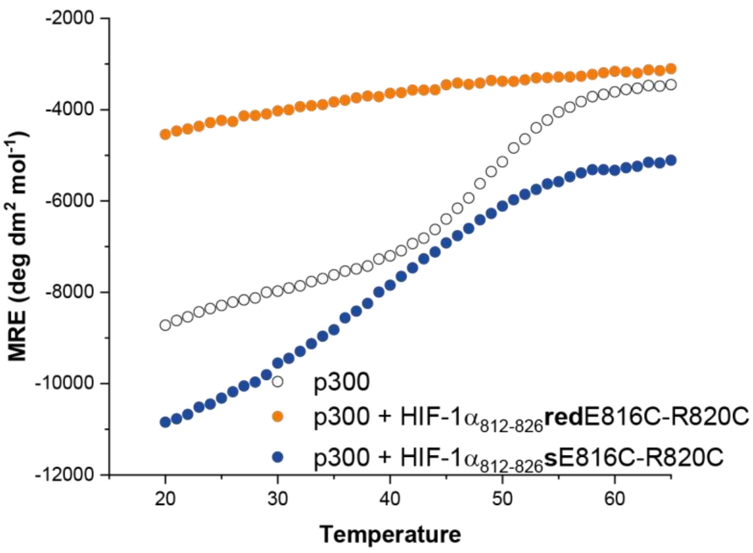

Figure S6. CD spectra for wt-HIF-1α812-826, HIF-1α812-826**s**E816C-R820C, HIF1α812-826**ox**E816C-R820C affected by the presence of p300 protein (20µM, 20 mM sodium potassium phosphate, pH 7.55) with stoichiometric peptides: HIF-1α812-826**s**E816C-R820C or HIF-1α812-826**red**E816C-R820C at 222 nm.

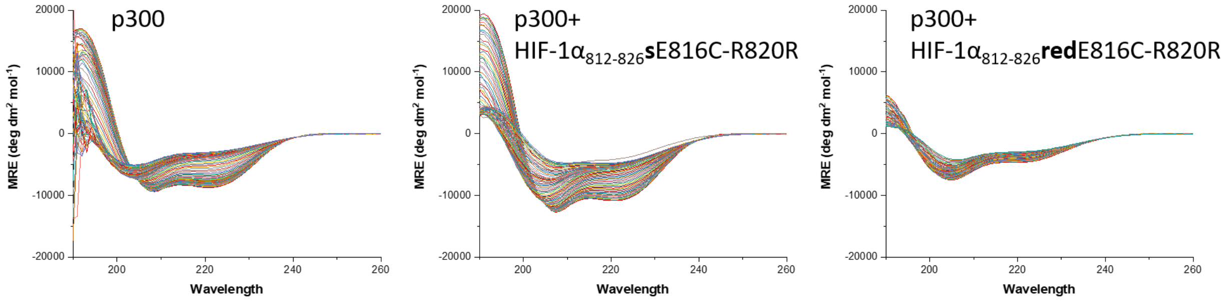

Figure S7. CD thermal melting experiment data of peptides/p300 in a 1:1 ratio at 250 µM concentration, in 20 mM Phosphate, pH 7.55

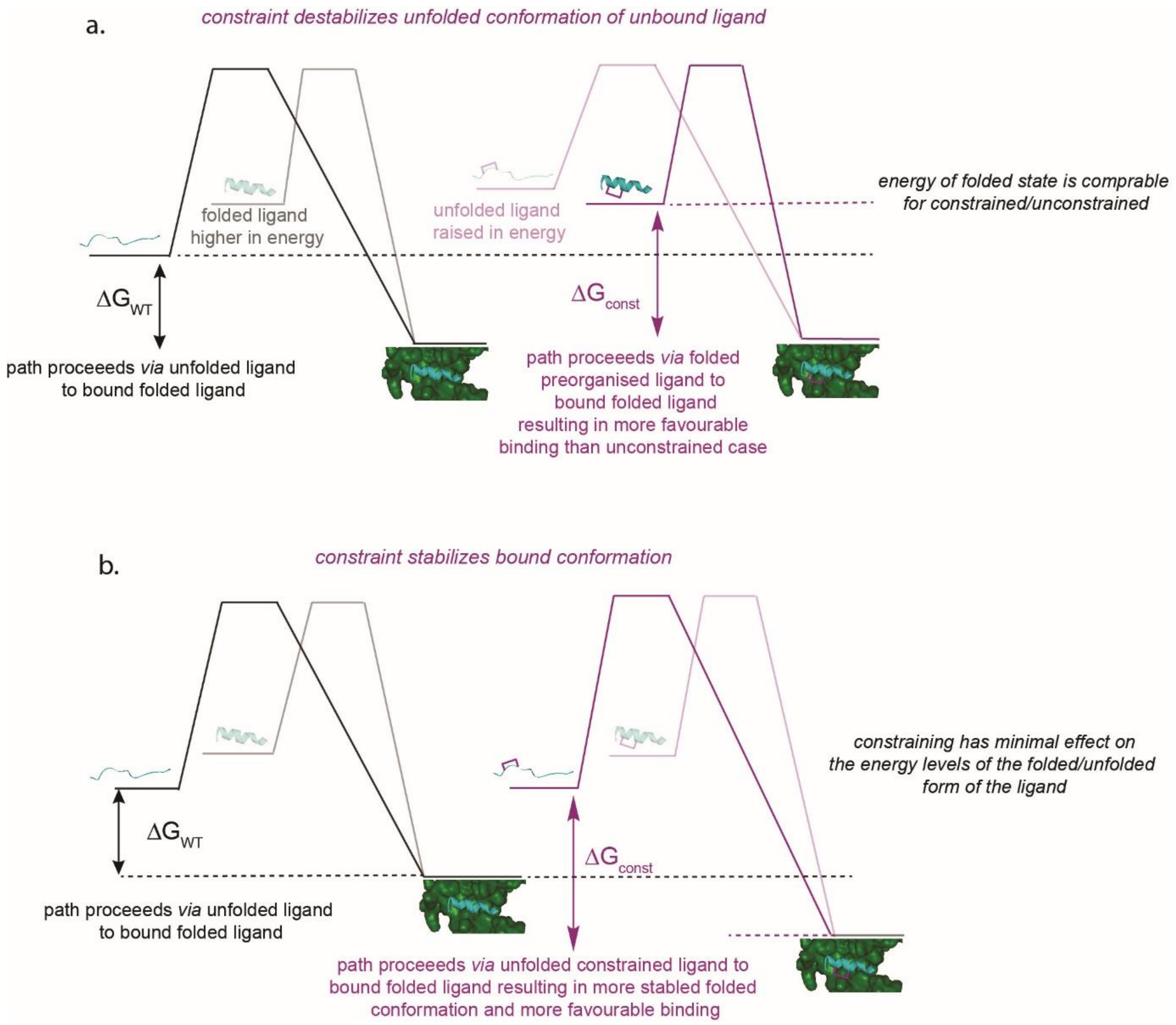

Figure S8. Schematic illustrating potential thermodynamic consequences of constraining a peptide in a bioactive helical conformation (a) constraining a peptide in a bioactive conformation destabilises the unfolded state rendering the folded conformer the lowest energy conformer which can lead to enhanced protein-binding affinity in comparison to a wild-type sequence. (b) constraining a peptide has no effect on the conformation of the unbound peptide, but leads to stabilisation of the bound conformation. A real experimental system is likely to have contributions from changes to both the energy of the unbound peptide and the bound peptide ligand as a result of introducing a constraint, whilst the pathway (and activation energies) are likely to be affected (not considered here). Similarly, differential conformational changes in the protein target (between unconstrained/constrained) and interactions between protein and constraint may also have a bearing on the thermodynamic landscape.

Peptide Characterisation Data

Tabulated HRMS data of synthesised peptides are shown below. Peptide identity was confirmed by the inspection of multiple charge states and are quoted as the monoisotopic peak for the Expected (Exp[d]) and Observed (Obs[d]) masses. Purity of peptides higher than 90% by analytical HPLC.

Peptide	Sequence	$[M+2H]^{2+}$ Exp[d]	$[M+2H]^{2+}$ Obs[d]
wt-HIF-1α$_{812-826}$	Ac-LQGEELLRALDQVN-NH$_2$	820.39	820.41
HIF-1α$_{812-826}$ **s**E816C-R820C	Ac-LQG**C**ELL**C**ALDQVN-NH$_2$ 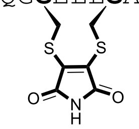	826.80	826.84
HIF-1α$_{812-826}$ **ox**E816C-R820C	Ac-LQG**C**ELL**C**ALDQVN-NH$_2$ 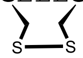	779.40	779.37
HIF-1α$_{812-826}$ **red**E816C-R820C	Ac-LQG**C**ELL**C**ALDQVN-NH$_2$ 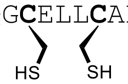	780.37	780.36
HIF-1α$_{812-826}$ **s**E817C-A821C	Ac-LQGE**C**LLR**C**LDQVN-NH$_2$ 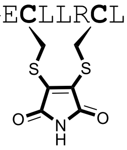	869.47	869.40
HIF-1α$_{812-826}$ **ox**E817C-A821C	Ac-LQGE**C**LLR**C**LDQVN-NH$_2$ 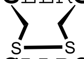	821.89	821.90
HIF-1α$_{812-826}$ **red**E817C-A821C	Ac-LQGE**C**LLR**C**LDQVN-NH$_2$ 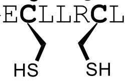	822.94	822.89

Peptide Analytical Data:

wt- HIF-1α$_{812-826}$

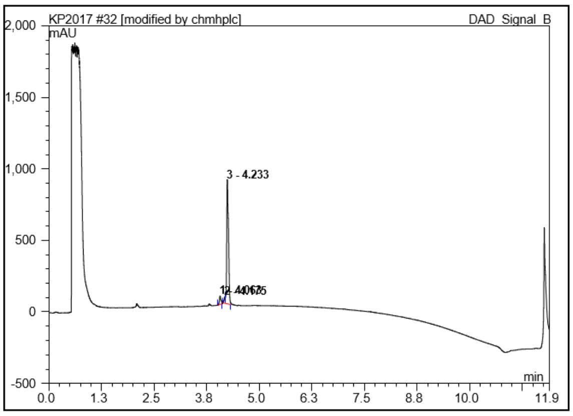

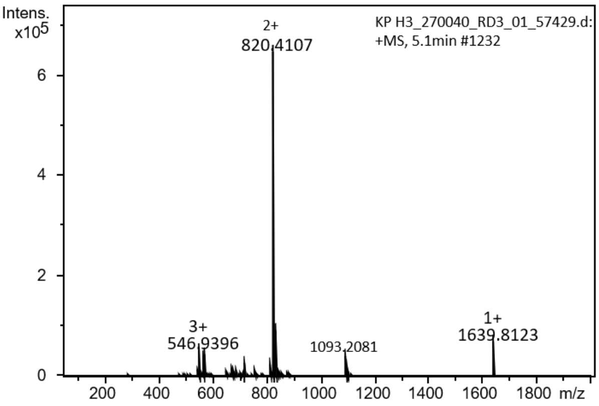

HIF-1α$_{SE816R820}$

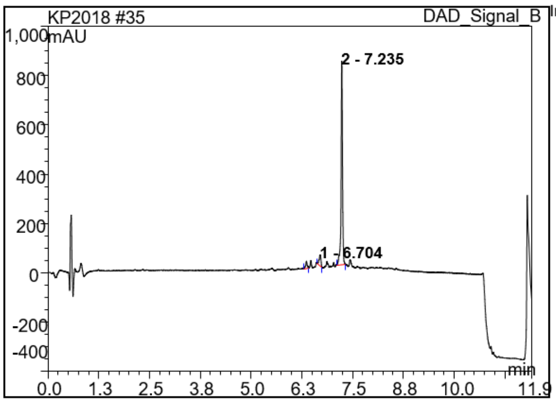

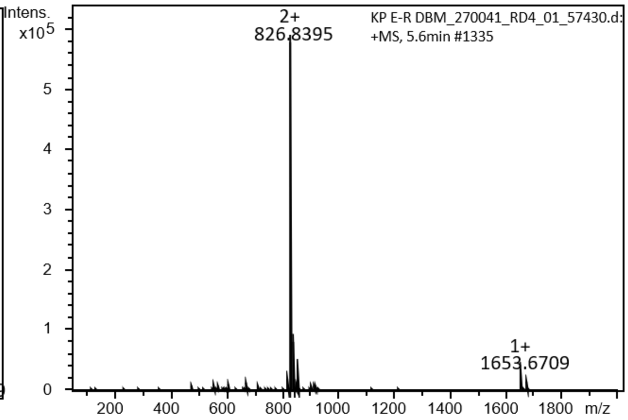

HIF-1α$_{RedE816R820}$

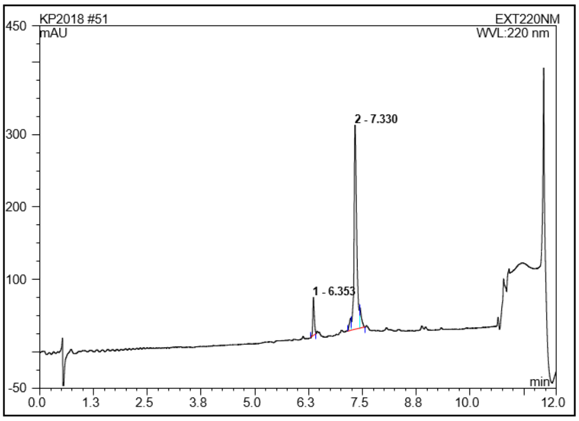

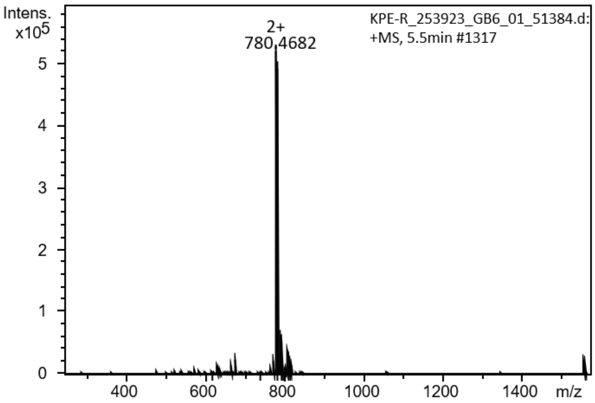

HIF-1α$_{OxE816R820}$

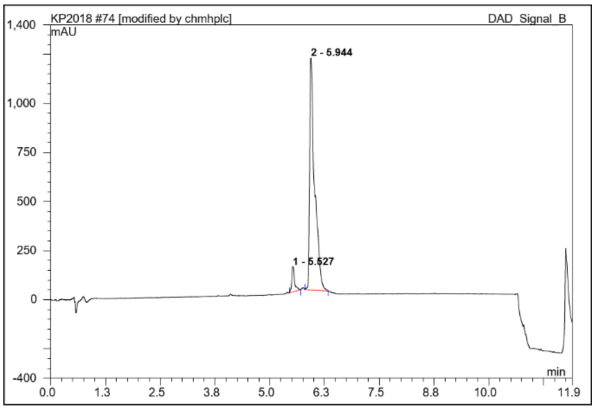

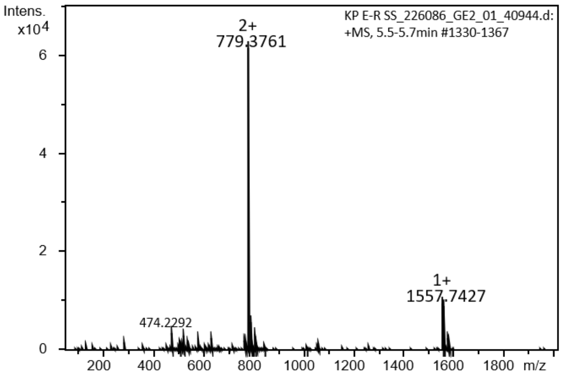

HIF-1$\alpha_{SE817A821}$

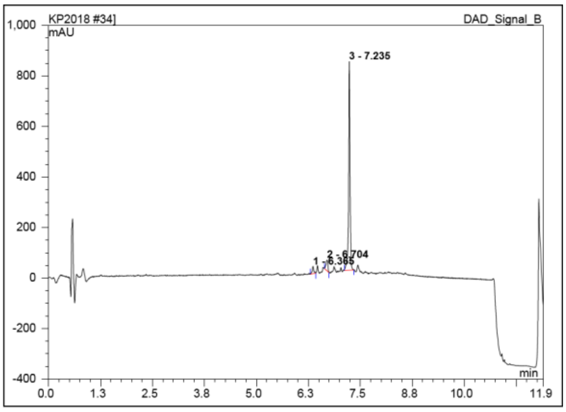

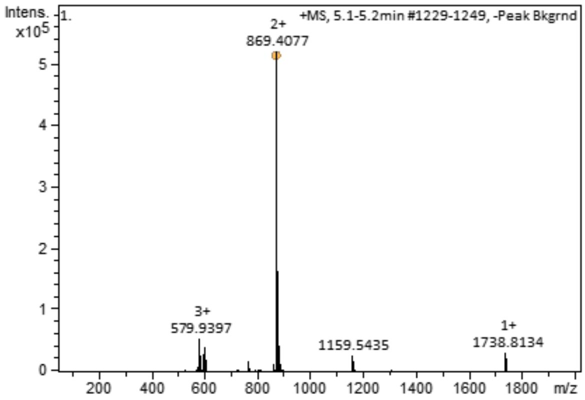

HIF-1$\alpha_{RedE817A821}$

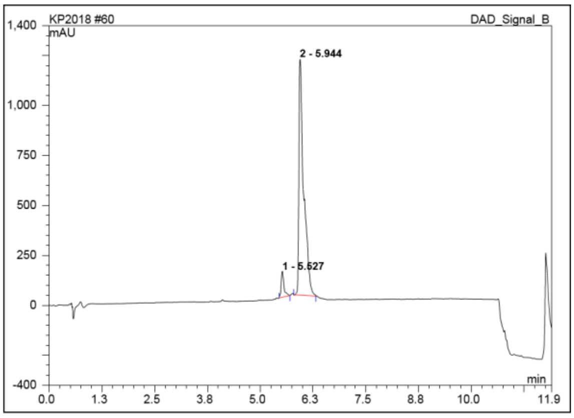

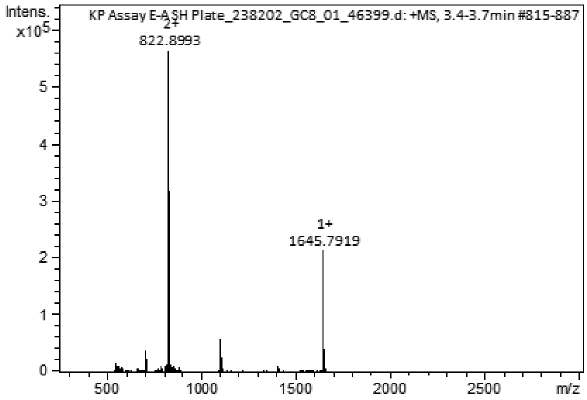

HIF-1$\alpha_{OxE817A821}$

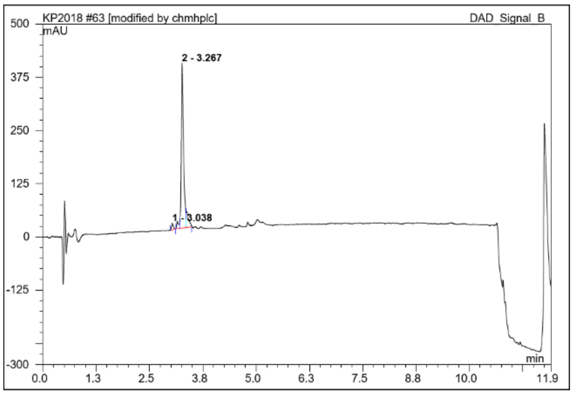

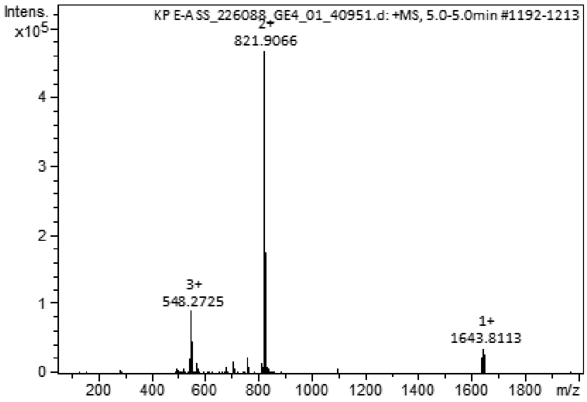

Solid phase peptide synthesis

For the microwave methods used, the temperature and total time is shown below:

Deprotection Microwave Methods

Method	Ramp Time	Total Time	Max Temp
Standard	20-30 sec	1:05	90 ºC
75 ºC deprotection*	30 sec	0:30	~50 ºC
	30-75 sec	3:00	75 ºC
Conventional*	N/A	5:00	rt
	N/A	10:00	rt

Coupling Methods

Method	Ramp Time	Total Time	Max Temp
Standard	20-30 sec	1:05	90 ºC
50 ºC MW	N/A	2:00	rt
	30-75 sec	4:00	50 ºC
Arg* coupling	N/A	25:00	rt
	30-75 sec	2:00	75 ºC
75 ºC coupling	30 sec	0:30	~50 ºC
	30-75 sec	5:00	75 ºC

*Methods for double deprotection/coupling
